# Increased Dietary Leucine Reduces Doxorubicin-Associated Cardiac Dysfunction in Rats

**DOI:** 10.3389/fphys.2017.01042

**Published:** 2018-01-17

**Authors:** Thiago M. Fidale, Hanna K. M. Antunes, Luciano Alex dos Santos, Fernanda Rodrigues de Souza, Simone R. Deconte, Francyelle Borges Rosa de Moura, Matheus M. Mantovani, Poliana Rodrigues Alves Duarte, Leonardo Roever, Elmiro S. Resende

**Affiliations:** ^1^Laboratory of Experimental Medicine, Federal University of Uberlândia, Uberlândia, Brazil; ^2^Department of Bioscience, Federal University of São Paulo, São Paulo, Brazil; ^3^Institute of Biomedical Sciences, Federal University of Uberlândia, Uberlândia, Brazil; ^4^Veterinarian Hospital, Federal University of Uberlândia, Uberlândia, Brazil

**Keywords:** doxorubicin, cardiotoxicity, leucine, BCAA, heart failure

## Abstract

Cardiotoxicity is one of the most significant adverse effects of the oncologic treatment with doxorubicin, which is responsible for a substantial morbid and mortality. The occurrence of heart failure with ventricular dysfunction may lead to severe cardiomyopathy and ultimately to death. Studies have focused on the effects of leucine supplementation as a strategy to minimize or revert the clinical condition of induced proteolysis by several clinical onsets. However, the impact of leucine supplementation in heart failure induced by doxorubicin is unknown. Therefore, the objective of this work is to evaluate the effects of leucine supplementation on the cardiotoxicity in the heart of rats treated with doxorubicin. Rats treated with a 7.5 mg/kg cumulative dose of doxorubicin for 14 days presented a dilatation of the left ventricle (LV), and a reduction of the ejection fraction (FE). The 5% supplementation of leucine in the rats' food prevented the malfunctioning of the LV when administered with doxorubicin. Some alterations in the extracellular matrix remodeling were confirmed by the increase of collagen fibers in the doxorubicin group, which did not increase when the treatment was associated with leucine supplementation. Leucine attenuates heart failure in this experimental model with doxorubicin. Such protection is followed by the maintenance of interstitial collagen fibers.

## Introduction

Doxorubicin is an antineoplastic agent from the group of anthracyclines, commonly used in a wide range of neoplasms treatment (Adão et al., 2013). Cardiotoxicity is one of the most meaningful adverse effects of oncologic treatments causing considerable morbidity and mortality (Raschi et al., 2010). Among the effects of chemotherapeutic agents on the cardiovascular system, cardiomyopathy, being highly frequent and serious, is the most prominent leading to heart failure with severe ventricular dysfunction and death (Takemura and Fujiwara, 2007; Albini et al., 2010). Evidence show that dysfunctions in the left ventricle are closely related to the use of cumulative doses of anthracyclines (Oliveira et al., 2017). Multiple mechanisms are involved in heart failure induced by doxorubicin. The main hypothesis for its cardiotoxicity is the increase of oxidative stress. Repeated administration can lead to permanent lesions at both cellular and interstitial levels, often associated to degeneration and cardiac muscle cells decrease, loss of contractile elements and heart failure (Octavia et al., 2012). Other cardiotoxic mechanisms that are induced by anthracyclines are related to compromising of important transcription synthesis factors involved in the regulation of cardiac-specific genes (Gianni et al., 2008). This decrease in the protein expression coupled with the increase degradation of myofilaments leads to a negative balance of sarcomere proteins in cardiac cells (Jeyaseelan et al., 1997), such as interaction decrease between actin and myosin filaments and disorganization of the sarcomere myosin (Bottone et al., 1998).

Efforts to reduce cardiotoxicity include alterations in the dexamethasone molecule and/or reduction of the therapeutic dose exposure. Alternatively, myocardial uptake of dexamethasone was reduced after the introduction of liposome encapsulation and the simultaneous administration of the iron chelator, dexrazoxane was employed to minimize reactive oxygen formation (Batist, 2007; Kaiserova et al., 2007). In addition to their potential translational benefit, these later strategies provided mechanistic insight into doxorubicin-linked cardiotoxicity (Sawyer et al., 2010; Adão et al., 2013).

The regulation of the metabolism, hypertrophy and cell apoptosis in heart failure is complex, but evidence suggests that these processes can be modulated by vital macronutrients in the diet, including amino acids (Layman, 2003; Kim et al., 2011). Leucine is an amino acid of branched and aliphatic chain, and studies have focused on the effects of the supplementation of this amino acid as a strategy to soften or/and revert the proteolysis in different clinical situations, such as hyperthyroidism (Fidale et al., 2016), such as rats treated with dexamethasone (Shah et al., 2000) and others with tumors (Ventrucci et al., 2001), which demonstrates that leucine supplementation was efficient in softening this damage. There is evidence that leucine has a direct anabolic effect over protein turnover in ischemic hearts, although there is a reduction in all circulating BCAAs in humans after a myocardial infarction. The greatest percentage leucine decrease happens in the circulation on the first day after a myocardial infarction before returning to a baseline level in three days (Szpetnar et al., 2004).

Many of the underlying mechanisms of LV dysfunction and induced by doxorubicin, are shared by other cardiac insufficiency forms. Studies using rats show that leucine reduced arrhythmia and heart failure in experimental myocardial ischemia, and can serve as an alternative energetic substrate, by providing metabolic intermediaries directly to the tricarboxylic acid (Marazzi et al., 2008). Therefore, the objective of this study is to evaluate the effects of leucine supplementation on the cardiotoxicity in the heart of Wistar rats treated with doxorubicin.

## Materials and methods

Thirty-six Wistar male rats, 2–3 months old, and weighing 260 ± 14 g were used (Supplementary Table 1). All animals were provided by the Animal Experimentation Biotechnology Center of the Federal University of Uberlândia (CEBEA-UFU) and had free access to feed and water. The monitoring boxes and bottles of water were exchanged for sterilized material twice a week, according to CEBEA's protocol. The feed was changed at the same frequency and the leftovers were discarded. All animal handling and exchanges were done in a BS60 BioSafety Cabinet (Tecniplast®) airflow biosafety cabinet. The present study was approved by the Ethics Committee on the Use of Animals (CEUA-UFU) under the registration number 115/14, on 12/15/2014.

### Leucine supplementation

Diets were offered for an adaptation period of 14 days before the doxorubicin treatment. During the 14 days of treatment, and for a 14-days period after the last injection of doxorubicin, adding up to 42 days of diet. Composition of the standard diet (SD) included a minimum concentration of 1.54 g/100 g (1.5%) leucine, according to the American Institute of Nutrition (AIN-93G) (Reeves et al., 1993) and it was provided to both control and DOXO groups. The leucine and leucine+DOXO groups were treated with a leucine rich diet (LRD) which consisted of SD supplemented with 5.0 g/100 g leucine (6.5% total), as used previously by Witham et al. (2013).

### Treatment with doxorubicin

For the treatment, 10 rats of the DOXO group received, during 2 weeks and three times a week, intraperitoneal injections of doxorubicin hydrochloride reaching the 7.5 mg/Kg cumulative dose, as proposed in previous study (Campos et al., 2011). Another group of 10 animals (Leucine+DOXO group) was associated with this dosage regimen, and they received, 2 weeks before the doxorubicin treatment, a 5% leucine supplementation in their food. A group of 8 animals, (control group), with SD, and another group of 8 animals, (Leucine group), with LRD, received a similar dose of saline solution for the same regimen used for the animals treated with doxorubicin.

### Echocardiography study

*In vivo* cardiac function was evaluated via Transthoracic Echocardiography 14 days after the end of the doxorubicin treatment. All animals were anesthetized using 0.1 ml/100 g Ketamine (10%) associated with the same dose of xylazine (2%). Anterior thorax trichotomy was performed, and the animals were put in 45° lateral decubitus. ESAOTE MyLab 30 VET gold and a 8.0 MHz transducer of 3.0 cm depth with a sectoral angle of 75° were used. In M mode, to the transversal right parasternal cut in the plan of chordae tendineae, of the left ventricle end-diastole diameter (LVEDD) and left ventricle end-systole diameter (LVEDS) was measured, for posterior calculation of the shortening fraction (%FS). In the same cut, the diastolic and systolic volumes were calculated by the Teichholz et al. (1976) method to the posterior calculus of the ejection fraction (%LVEF).

### Histopathological analysis

Each heart was divided into four pieces (base, middle proximal, distal middle and apex) by making three transverse cuts. These pieces were fixed in a 10% formalin solution for 48 h. Material processing followed the stages of dehydration, diaphanization, bath and embedding in paraffin. Next, the middle proximal segment was sectioned (5 um) and then stained for collagen using Picrosirius Red. The histological cuts stained in Sirius red were analyzed in the optical microscope *Nikon ECLIPSE TI-S*® and photographically registered (40x objective, 10x ocular). Each cut had a photographical register of at least 10 fields of the free wall of the left ventricle.

After obtaining the photomicrographs, the quantification of the total collagen area, given in percentage, was obtained with the aid of the threshold tool of the software ImageJ1.6.0_24. This tool allows limiting the percentage of collagen area deposited in the extracellular matrix in relation to the total area of the evaluated field.

### Statistical analysis

The experimental results were expressed as mean ± standard deviation and the normality of the data was tested using the Shapiro-Wilk test. For the normal data, the comparisons among groups was done through analysis of variance (ANOVA), and after that, a Tukey's test or Newman-Keuls Multiple Comparison Test was performed, just in case. Statistical analysis was done with a GraphPad statistical package Prism (5.0 version). Statistical significance was established when the value of *p* < 0.05.

## Results

### General observations

No deaths occurred in any of the groups during the experiment. The animals treated with doxorubicin showed less movement and developed periocular and nasal exudation, as well as reddened lesions in the legs similar to drug dermatitis.

### Effects of elevated dietary leucine on left ventricular dysfunction induced by doxorubicin in wistar rats

**Figure 1:**
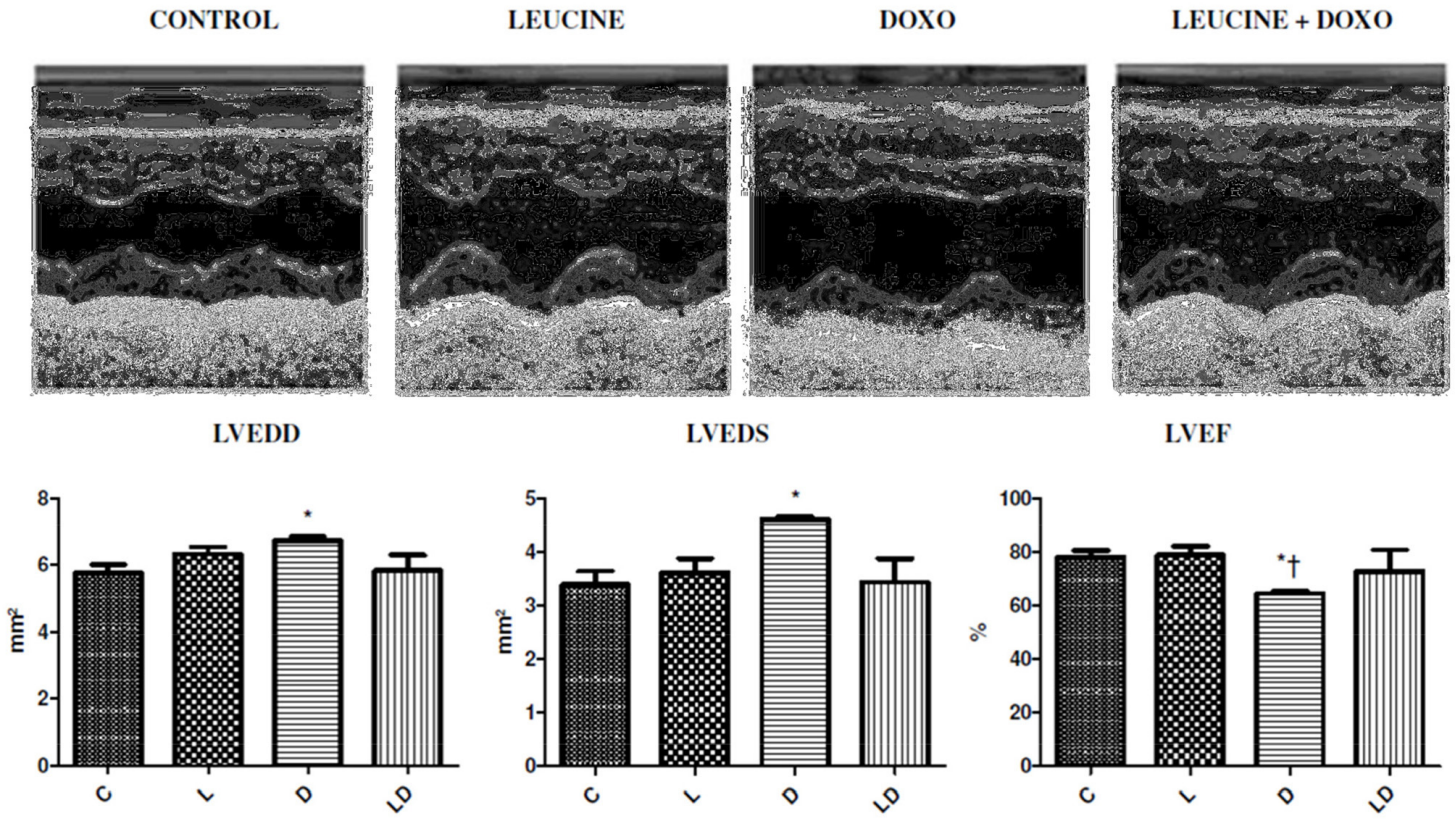
Leucine prevents ventricular dilation and reduces the ejection fraction produced by doxorubicin. Echocardiographic images with dilation and reduction of LV ejection fraction induced by doxorubicin and protected leucine products in the leucine+DOXO group. ^*^p < 0.05 in relation to the Control Group (ANOVA), ^†^*p* < 0.05 in relation to the Leucine Group (ANOVA). C, Control Group; L, Leucine Group; D, DOXO Group; DL, DOXO+Leucine Group.

Firstly, the protector role of the leucine induced cardiotoxicity by doxorubicin in rats was tested. In order to achieve this purpose, 2 groups of 10 rats were injected with the 10-mg/kg cumulative doses during 2 weeks, one of the groups also received in its diet a 5% leucine supplementation. Fourteen days past the final treatment, DOXO group presented a higher diameter of the left ventricle at the end of systole (LVEDS) 4.61 ± 0.1 mm, and at the end of diastole (LVEDD) 6.72 ± 0.3 mm, when compared to Control group (LVEDS) 3.39 ± 0.7 mm (*p* = 0.0132) and (LVEDD) 5.76 ± 0.7 mm (*p* = 0.045). The ejection fraction of DOXO group was (LVEF) 64.5 ± 2%, which is 17% lower than the control group (LVEF) 78.0 ± 7% (*p* = 0.0153). In groups leucine+DOXO, diameters of left ventricle were lowest (LVEDD) 5.82 ± 1.0 mm, (LVEDS) 3.44 ± 0.9 mm, and the ejection fraction (LVEF) 72.8 ± 17% was higher than DOXO group, however they did not show significant differences concerning both control and DOXO groups, as shown in Figure 1.

### Leucine effect on the remodeling of the extracellular matrix induced by the treatment with doxorubicin

**Figure 2:**
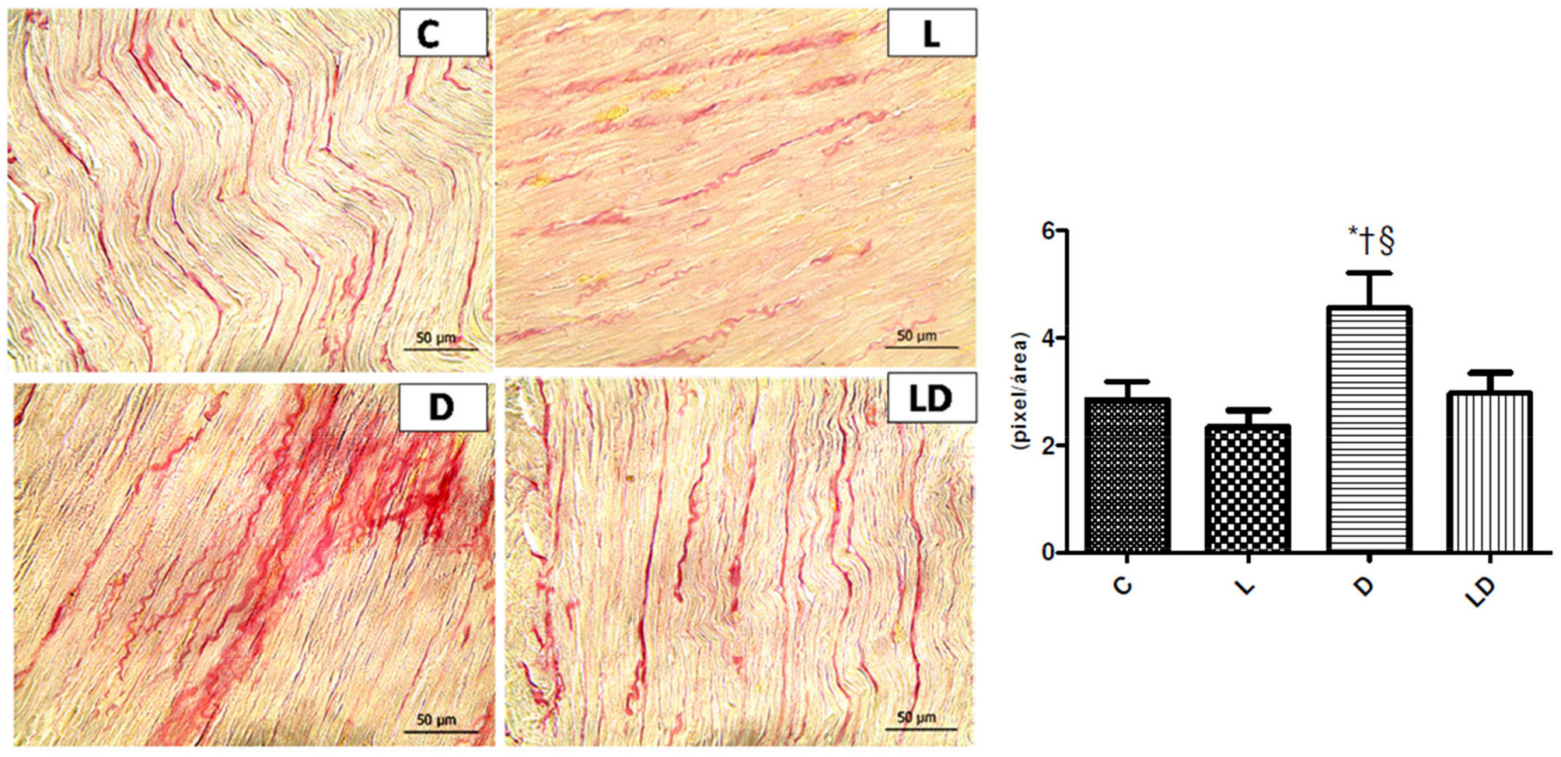
Leucine preserves cardiac structure and increase of collagen fibers produced by doxorubicin. Samples of histopathological images with increased amount of collagen fibers in the extracellular matrix induced by doxorubicin and the protective effects of leucine in the leucine+DOXO group. ^*^p < 0.05 in relation to Control group (ANOVA); ^†^*p* < 0.05 in relation to Leucine group (ANOVA); ^§^*p* < 0.05 in relation to the leucine+doxo group (ANOVA). C, Control Group; L, Leucine Group; D, DOXO Group; DL, DOXO+Leucine Group.

Changes in the extracellular matrix remodeling were confirmed by a greater quantity of collagen fibers in the DOXO group 4.55 ± 2.6 mg/mg, when compared to the control group 2.85 ± 1.3 mg/mg (*p* = 0.0270) and to the leucine+DOXO group 2.97 ± 1.5 mg/mg (*p* = 0.0141), according to Figure 2.

## Discussion

Doxorubicin is used in antineoplasic therapies, yet it can cause left ventricular dysfunction and heart failure (Raschi et al., 2010; Adão et al., 2013). In this experimental model, as far as we know, the data show for the first time that leucine is capable of reducing the effects of the cardiotoxicity of doxorubicin. Beneficial effects were followed by the maintenance of cardiac function and cardiac collagen fibers.

Cardiotoxicity related to doxorubicin is a multifactorial process, sustained by energy collapse, disturbance of homeostasis and suppression of specific sarcomere cardiac genes, apoptosis of cardiomyocytes followed by remodeling and dilated cardiomyopathy (Minotti et al., 2004). The anthracyclines also induce cardiac dysfunction because of calcium metabolism alterations, malfunction of the sarcoplasmic reticulum pump (SERCA), of the phospholamban (PLB) and improper opening of ryanodine receptors (RyR2) (Lim et al., 2004; Whitehead et al., 2006).

The results of the current study are in agreement with results of previous studies (Campos et al., 2011) that found a significant reduction in left ventricular ejection fraction in the group of rats treated with a cumulative dose of 15 mg/Kg of doxorubicin. The ejection fraction was lower than the control seven days after the last injection. And the groups treated with 3.75 mg and 7 mg/kg presented a significant reduction in ejection fraction and shortening fraction in relation to the control 14 days after the last injection. The results showed that the cardiac toxicity of doxorubicin was effectively attenuated by elevated dietary leucine. Maintenance of cardiac function was observed in our data in the leucine+doxorubicin group compared to its control, whereas the doxorubicin group alone presented reduction of the ejection fraction when compared to the control group.

Rats treated with different cumulative doses of doxorubicin, administered over 8 weeks, totaling 8, 12, and 16 mg/kg had a higher area of fibrosis in the animals' heart when compared to the animals in the control group (Oliveira et al., 2017). We observed the structural preservation of the rat heart in the leucine+doxorubicin group, maintaining the amount of collagen fibers interstitial compared to the doxorubicin group alone.

An earlier study (Ito et al., 1990) observed in a rat cardiac muscle cell culture model that treatment with doxorubicin resulted in a rapid and selective decrease in the expression of specific cardiac genes. Doxorubicin selectively decreased mRNA levels for the sarcomeric, a-actin, troponin I and myosin light chain genes. These changes in cardiac muscle precede ultrastructural changes associated with myofibrillar functional losses, such as decreased shortening fraction and ejection fraction, according to our findings. They also observed the effect of different concentrations 0.2 and 0.5 μM of doxorubicin in protein synthesis in cardiac cell culture after incorporation of leucine into the culture for 4 h. After 24 h of these treatments protein synthesis stimulated by leucine was inhibited by 41 and 68%, respectively.

Previous studies have pointed out to cardiac protection with leucine as a therapy strategy in different models of systolic dysfunction, suggesting that leucine acts as a nutritional sign to stimulate protein synthesis in cardiac muscle, increasing the availability of the eukaryotic initiation factor (eIF4B) and activating via p70S6K and mTOR cardiac, a fact that can have attenuated damages observed in the doxorubicin group (Escobar et al., 2006; Canedo et al., 2010). In an experimental hyperthyroidism model, a significant reduction of serum CK-MB was observed in a group of rats supplemented with 5% leucine through the food, in comparison to a group with isolated hyperthyroidism, suggesting a cardioprotective effect of this amino acid by proteic synthesis modulation (Fidale et al., 2013). Nevertheless, the clinical implication of this fact are unknown and the eventual relation with leucine mechanism is of speculative nature. In this experimental model, leucine attenuates left ventricular dysfunction with doxorubicin and such protection is followed by maintenance of interstitial collagen fibers.

Leucine may protect against cardiotoxicity by preventing the loss of post-translational efficiency and/or developmental autophagy. Both of the efficiency and onset of autophagy are postulated to develop in response to doxorubicin, and are putative contributors to the cardiotoxicity.

### Study limitations

Our study has some limitations. Doxorubicin is administered in patients with cancer, while our study investigates experimental cardiotoxicity in rats that did not have this disease. This study design may have influenced our results, despite the focus of our work being the specific identification of the effect of leucine in heart failure and quantity of collagen fiber induced by doxorubicin. Leucine may not adequately protect against cardiotoxicity when doxorubicin is administered at levels that produce cancer-related lethality, moreover, the present study did not monitor the consumption of food and water. Another aspect is that this study did not aim at identifying the possible pathways of doxorubicin in which leucine may have acted and improved the animals' cardiac conditions. In this sense, new studies should be conducted, in order to map protein synthesis pathways and cellular death, aiming at identifying possible interventions of leucine in cardiac cells.

The design of the study may have influenced our results, although the focus of our work was the specific identification of the leucine effect on heart failure and the amount of collagen fiber induced by doxorubicin. Another aspect is that this study did not aim to identify the possible pathways of action of doxorubicin in which leucine may have acted and improved the cardiac conditions of the animals. In this sense, new studies must be carried out in order to map pathways of protein synthesis and cell death, in order to identify possible leucine interventions in cardiac cells.

## Author contributions

All authors listed have made a substantial, direct and intellectual contribution to the work, and approved it for publication.

### Conflict of interest statement

The authors declare that the research was conducted in the absence of any commercial or financial relationships that could be construed as a potential conflict of interest.
