# Supplementary material for: Increased Dietary Leucine Reduces Doxorubicin-Associated Cardiac Dysfunction in Rats
Source: Front Physiol. 2018 Jan 17;8:1042. doi: 10.3389/fphys.2017.01042 (PMC5779071; doi:10.3389/fphys.2017.01042)
Supplement: Supplementary file 1 [file Table1.docx]

**Supplementary Information**

Additional Information from the present study can be found in the table 1.

**TABLE 1 – Analyzed variables**

| **Analyzed Variables** | | **Control** | **Leucine** | **Doxo** | **Leucine + doxo** |
| --- | --- | --- | --- | --- | --- |
| **Anthropometric**  **Variables** | Initial BW (g) | 259,5±27 | 281,9±10 | 251,6±38 | 251,8±14 |
|  | Final BW (g) | 434,9±37 | 432,4±49 | 363,7±38*† | 356,1±35*† |
|  | BH (g) | 1,04±0,12 | 0,97±0,07 | 0,90±0,18 | 0,95±0,21 |
|  | BH/TL (mg/cm) | 26,67±3,34 | 24,50±1,85 | 24,29±5,12 | 25,63±5,65 |
| **Echocardiography** | FC (bpm) | 345,2±50 | 311,7±57 | 312,4±24 | 315,4±28 |
|  | FS (%) | 42,5±8 | 43,5±9 | 30,7±2† | 40,2±15 |
|  | LVEF (%) | 78,0±7 | 78,8±9 | 64,5±2†* | 72,8±17 |
|  | LVEDS (mm²) | 3,39±0,7 | 3,61±0,8 | 4,61±0,1* | 3,44±0,9 |
|  | LVEDD (mm²) | 5,76±0,7 | 6,32±0,6 | 6,72±0,3* | 5,82±1,0 |
| **Histopathology** | Collagen (mg/mg) | 2,85±1,3 | 2,34±1,3 | 4,55±2,6*†§ | 2,97±1,5 |
| **Feed consumption** | FC (g) | 34±7 | 36±6 | 33±2 | 32±2 |

***Table 1*** *- Values expressed in average ± standard deviation for: BW: body weight; BH: heart absolute weight; TL: tibia length; FC: heart rate; %FS: shortening fraction of the left ventricle; %LVEF: ejection fraction of the left ventricle; LVEDS: diameter of the left ventricle during systole; LVEDD: diameter of the left ventricle during diastole;FC: daily feed intake per animal. *p<0,05 in relation to Control group (ANOVA); †p<0,05 in relation to Leucine group (ANOVA); §p <0.05 in relation to the leucine + doxo group (ANOVA).*
